# Influence of Different Polymer Types on the Overall Release Mechanism in Hydrophilic Matrix Tablets

**DOI:** 10.3390/molecules14082699

**Published:** 2009-07-24

**Authors:** Anna Körner, Lennart Piculell, Frida Iselau, Bengt Wittgren, Anette Larsson

**Affiliations:** 1 SCA Hygiene Products, SE-405 03 Göteborg, Sweden; E-mail: anna.korner@sca.com (A.K.); 2 Department of Physical Chemistry 1, Lund University, Box 124, SE-221 00, Lund, Sweden; E-mail: lennart.piculell@fkem1.lu.se (L.P.); 3 AstraZeneca R&D, SE-431 83, Mölndal, Sweden; E-mails: frida.iselau@astrazeneca.com (F.I.), bengt.wittgren@astrazeneca.com (B.W.); 4 Department of Chemistry and Bioengineering, Chalmers University of Technology, SE-412 96, Gothenburg, Sweden

**Keywords:** hydrophilic matrix tablets, release mechanism, intrinsic viscosity, PEO, HPMC, dextran

## Abstract

The effect of three different types of polymer chain structures on the polymer release from hydrophilic matrix tablets was investigated by comparing a synthetic semi-crystalline linear polymer (PEO), a branched amorphous polysaccharide (dextran) and an amorphous substituted cellulose derivative (HPMC). The polymer release rates for tablets containing mixtures of high and low molecular weight grades in different ratios were determined by using a modified USP II method and a SEC-RI chromatography system. The results showed that independent of polymer type: (i) plots of the release versus time had similar shapes, (ii) the release of long and short polymer chains was equal and no fractionation occurred during the release and (iii) the release rate could be related to the average intrinsic viscosity of the polymer mixtures. This confirms the hypothesis that the release rate can be related to a constant viscosity on the surface of the hydrophilic matrix tablet and that it is valid for all the investigated polymers.

## 1. Introduction

The use of hydrophilic matrix tablets as drug delivery systems started already during the 60s and is today one of the major extended drug release platforms [1,2,3]. Reasons for the large industrial interest and use of the hydrophilic matrix tablets is probably that this platform can be used for a variety of drugs with different preformulation properties, and furthermore, the production process is conventional and robust, leading to a relative low the production cost [4]. Different polymers can be used as matrix formers; for example polysaccharides like xanthan, dextran, or substituted cellulose derivatives like hydroxypropyl methyl cellulose (HPMC) and hydroxyethyl cellulose or synthetic polymers like poly(ethylene oxide) (PEO) and polyacrylic acid [5,6,7,8,9,10].

The drug release rate from hydrophilic matrix tablets is determined by several properties, such as the composition of the formulation, the manner of production, the properties of the drug itself and the properties of the polymers in the matrix, where molecular weight, hydrophilicity, degree of cross linking, degree of substitution, etc, are all polymer parameters known to influence the swelling and erosion of the matrices [4]. Many experimental studies and modelling efforts have been performed in order to reveal the detailed physics behind the dissolution process of a polymer. Much of this work is summarized in recent review articles [11,12]. However, these models often contain parameters that are not available from published experiments. Therefore, the models can only be used for qualitative comparisons. To understand the polymer release one needs to look into what happens when a dry hydrophilic polymer matrix tablet comes in contact with water. During this step a high-viscous gel layer of macroscopic thickness will develop and surround the solid core. This gel layer is a polymer solution of high concentration and it will remain stationary, even if the surrounding solution is stirred. When a fluid passes an ordinary solid surface a velocity gradient is formed in the fluid due to the shear forces causing the fluid to slow down to zero velocity at the dry solid interface. Also, when a flowing fluid passes the surface of a dissolving hydrophilic matrix tablet the shear forces cause the fluid to slow down. However, the surface of the swollen tablet is not rigid; instead the surface is a very viscous polymer solution. A force balance is therefore established between the shear force developed by the external liquid and the resistance of the gel layer towards shear. Specifically, this force balance may be assumed to result in a certain threshold viscosity at the gel surface, and the part of the gel layer having a viscosity below the threshold value will be rapidly rinsed off from the tablet. This threshold viscosity can be represented by a critical polymer concentration, c_s_, at the gel/solution boundary, and for a given tablet c_s_ will be constant during the entire dissolution process when the shear environment remains constant. The viscosity depends on polymer parameters like molecular weight, hydrophilicy etc and the threshold viscosity on the gel surface of matrix tablets will correspond to different concentrations c_s_ depending of used matrix polymer.

These ideas behind the polymer release mechanism have been proposed by Ubereiter *et al*. [13] and Ju *et al*. [6] and have been further extended by us in a model in which we have shown that c_s_ can be related to the release rate dm/dt. The models starts in the phenomenological mass transfer equation [14,15]:

(1) $${\frac{dm\;}{dt}|}_{s}=k_{s}A_{s}c_{s}$$

where c_s_ is the critical polymer concentration on at the gel surface of the matrix, A_s_ is the area over which mass transfer occurs, k_s_ is a phenomenological rate constant, describing the rate at which the water penetrates into the gel and dilutes the polymer down to the critical value c_s_. The value of k_s_ is expected to depend on the gradient of the chemical potential of water (the solvent) in the outermost part of the gel layer. The release rate when 50 % of the polymer has been released, r_50_ , can be an estimate of (dm/dt)_50_ and this together with k_s_=k_s,50_ and A_s_=A_s,50_ gives:

(2) $$r_{50\;}=k_{s,50}A_{s,50}c_{s}$$

This equation was shown to be valid for PEO samples with different average molecular weights [14] and different polydisperties, where c_s_ was estimated from the assumption that c_s_ is direct proportional to the overlap concentration, c*, and that c* is inversely proportional to the intrinsic viscosity [η]. By using this relation and Equation 2 the release rate r_50_ can be expressed as:

(3) $$r_{50\;}\propto c*\propto 1/\left[\eta \right]$$

Another finding for PEO matrix tablets was that the release rates of short and long polymer chains were equal [7,14]. This means that no fractionation of polymer occurs during the release process within the gel layer. This result was explained by the fact that the relative movement of polymers chains within the gel layer was negligible and that the swelling and dilution of the gel layer by water was faster than the polymer-polymer movement. Thus, in a homogenous mixture of short and long PEO chains in the matrix the polymer chains will enter the gel-solution interface at the same time regardless of polymer size.

**Figure 1:**
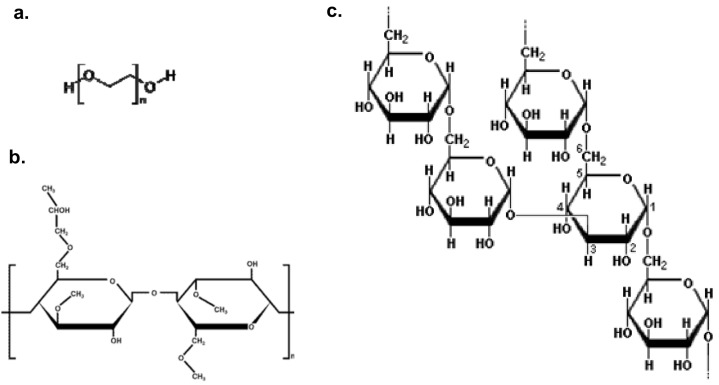
Schematic chemical structures for: (a) PEO, (b) HPMC and (c) dextran.

Since no systematic studies relating the polymer release rate to the intrinsic viscosity have been performed for hydrophilic matrix tablets other than PEO [7,14,16] one can ask if the model presented is also valid for other polymers. It is not obvious that this should be the case, since PEO is special in the sense that it is a synthetic semi-crystalline linear polymer. To test if the hypothesis is also valid for other types of polymer, two amorphous polysaccharides in the glassy state, dextran and hydroxypropyl methyl cellulose (HPMC) were chosen as model polymers (Figure 1). Furthermore, dextran is a branched polysaccharide available in many different molecular weights, whereas HPMC is a substituted cellulose derivative and the most used polymer in hydrophilic matrix tablets for extended drug release formulations. In order to test our hypothesis we needed to determine the rheological properties and release rates from matrixes of the chosen polymers and mixtures whereof. The results in this study showed that the proposed model was valid for all investigated polymers, but that the release mechanism contained more elements than was covered by the suggested model.

## 2. Results and Discussion

### 2.1. Characteristics of the polymer samples

Table 1 summarizes the measured number average molecular weight (M_n_), weight average molecular weight (M_w_), polydispersity index (PI) and intrinsic viscosity ([η]) of the polymers and polymer mixtures used in the study. For the convenience of the reader, we also include corresponding data for PEO, which have been published before [7,14,16]. In general, both the number-average and the weight-average molecular weights increase with increasing amount of high-molecular weight polymer in the sample. M_w_ is shown to be much more sensitive than M_n_ to the addition of small amounts of high molecular weight polymer, as would be expected from the definition of the two averages. Furthermore, for low molecular weights, the precision of the M_n_ measurements is known to be low, due to a low signal from the MALS-detector [17]. This explains why M_n_ for the samples in the dextran and HPMC series initially decrease with the addition of high molecular weight polymer. From the table it can also be seen that the polydispersity of the nonmixed commercial samples increases with increasing molecular weight.

### 2.2. Release experiments

When a tablet was exposed to the dissolution medium it soon developed a gel layer for all investigated types of polymers. However, the appearance of the gel layer was strikingly different for the different polymer types. For the dextran tablets the gel layer was very thin and the dimensions of the tablet decreased throughout the release experiment. Similar features have been observed for low molecular weight PEO tablets (M_w_ of about 10^5^) [14]. The time for total release of the various dextran tablets varied in the interval 2–5 h. For the HPMC tablets the gel layer was transparent and grew with time. It was noticed that the major growth took place in the axial direction. We have no explanation for this phenomenon, but it has been observed also in other studies [18,19].

**Figure 2:**
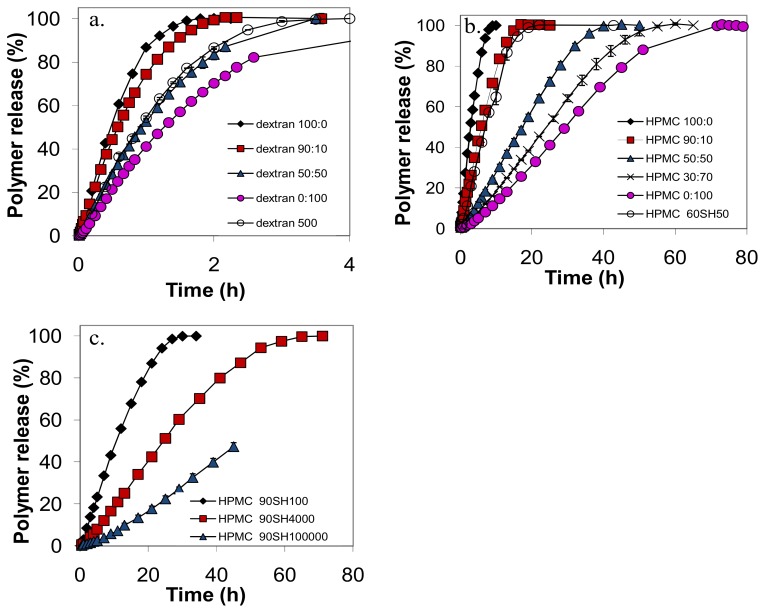
The release curves of: (a) dextran; (b) HPMC60SH and (c) HPMC 90SH as indicated by the figure legend. The symbols denote the calculated average from two tablets and the error bars indicates the positions of the individual measurements.

Figure 2 shows that the shapes of the release curves were smooth and similar for all the tablets in the study, with a slow initial release rate, an almost linear release rate during the main release period, and a slower release rate at the end of the experiment. This general shape, similar for nonmixed and mixed samples, was also observed for the release profiles of PEO tablets in previous work [7]. The release time, t_50_, defined as the time when 50% of the polymer in the tablet had been released, was taken directly from the release profile for each polymer. The release rate at 50% released amount polymer, r_50_, was taken as the slope of a linear fit to the experimental points between 10 and 60% released polymer. The r_50_ and t_50_ values for the various tablets in the study are summarized in Table 1.

### 2.3. Absence of fractionation of polymer from polymer mixtures during release

**Figure 3:**
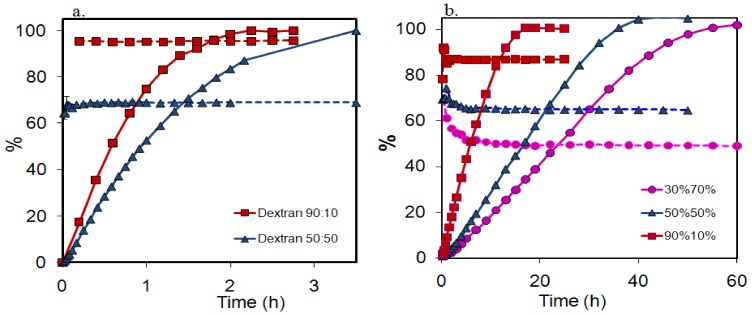
The released fraction low molecular weight polymer (dotted lines) together with the polymer release profiles (full line) for the mixed dextran compositions as indicated in the figure legend (a) and the mixed HPMC 60SH compositions as indicated in the figure legend (b). The symbols denote the calculated average from two tablets and the error bars indicates the positions of the individual measurements.

In Figure 3 the fractions of low molecular weight polymer in the release samples for the mixed dextran and HPMC 60SH tablets, respectively, are plotted against time, together with the release profiles which are also included in the plots. As observed previously in a similar analysis of mixed PEO tablets [14], the fraction of low molecular weight (LMW) polymer released from the tablets was constant during the main part of the release experiment. For the HPMC tablets, the LMW fraction was released at a slightly higher rate than the HMW fraction during a short initial time period, and the same feature was also observed in the previous PEO study. For the dextran tablets no initial fractionation could be observed.

**Figure 4:**
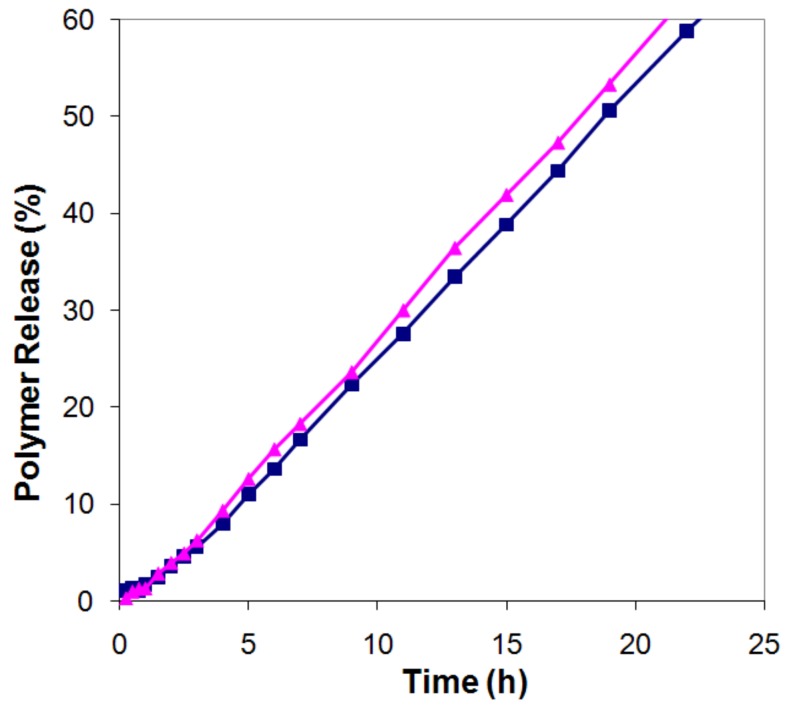
Release profiles up to 60% released polymer, for the low molecular fraction (triangles) and high molecular weight fraction (squares) of tablets from HPMC 50:50.

Since the data in Figure 3 refer to the accumulated LMW fraction in the release medium, they do not directly give information on the release rate of the LMW fraction as a function of time. In Figure 4 we have therefore converted the data for the HPMC 50:50 tablets into release profiles for the LMW and HMW fractions.

This conversion was made from the data in Figure 3b, by multiplying each point in the overall release profile with the fraction LMW (or HMW) polymer obtained at that point, and normalizing these data to 100% at full dissolution. It can be seen that the release rates (the slopes of the release curves) for the two fractions, within the experimental uncertainty, were identical (LMW: r_50_=3.05, HMW: r_50_=3.08, overall r_50_=3.06). The same was true for all compositions investigated in Figure 3.

Many theoretical models [11,12] assume that the polymer molecules are released from a swollen polymer by diffusion of the molecules through the gel layer (reptation), and that this step is the rate limiting step in the dissolution process. However, this picture is not supported by our results. According to the reptation theory, the diffusion constant should depend in the molecular weight as D∝M^-2^ [20]. Thus, if the polymer diffusion in the gel layer is the rate-limiting step, the diffusion of the shorter polymer molecules is expected to be much faster than the diffusion of the long molecules, and a pronounced fractionation would occur during the dissolution. According to the model proposed in our work, the release from the swollen gel layer is determined by a certain threshold viscosity at the tablet surface, and the polymer self-diffusion in the gel layer is negligibly slow, so that the mutual diffusion coefficient between polymer and water is determined by the water self-diffusion coefficient. Consequently, the polymer components do not move significantly relative to each other during the time it takes them to reach the threshold viscosity where the outermost part of the gel layer is rinsed off, and no fractionation should occur. Indeed, the experimental observations from the three different polymer systems support this model.

### 2.4. Release and swelling

**Figure 5:**
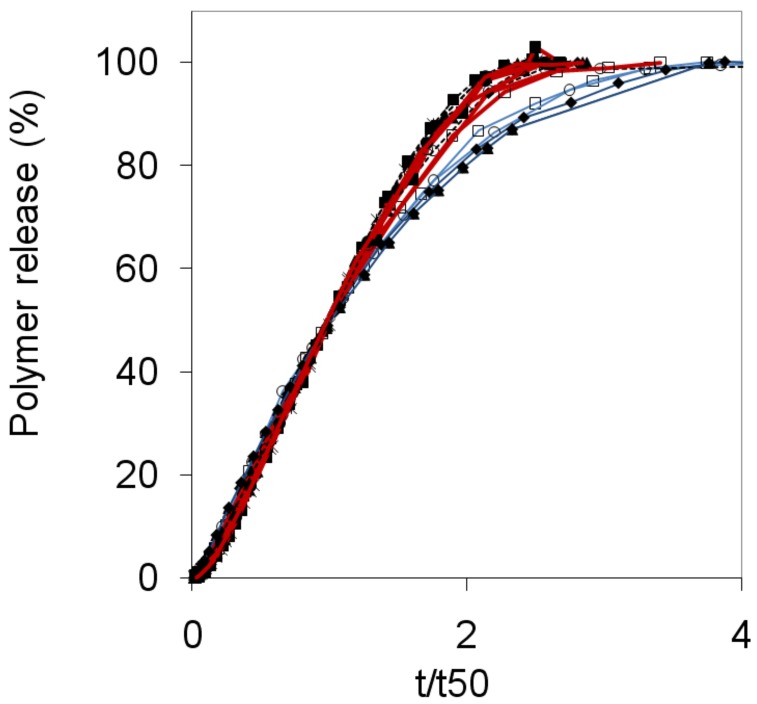
The polymer release is plotted against the reduced time (t/t_50_) for dextran (blue line), HPMC 60SH (black line) and PEO (red line, adopted from reference [7]) as indicated in the figure legend. 100:0 samples are represented by open squares, 90:10 by filled diamonds, 50:50 by filled triangles, 30:70 by filled squares, 0:100 by asterisks and the nonmixed samples by circles.

Figure 5 compares the release profiles of dextran, PEO and HPMC tablets on a reduced time scale, t/t_50_. This type of plot clearly illustrates the general similarity in shape of the release profiles for different mixtures, and also for different polymer types. However, there are significant deviations especially at times t > t_50_. Here the data fall into two main categories, with PEO and HPMC tablets in the first category and the dextran tablets in a second category, whose curves fall systematically below the other polymers. In general, the tablets in the first category display a significant swelling, whereas the dextran tablets have thin gel layers and thus the tablet dimensions decrease throughout the release experiment. Equation (3) predicts that the polymer release rate of a given tablet should depend explicitly on the area of the swollen tablet (A_s_). Such dependence was confirmed by detailed measurements of tablet swelling as a function of time [16]. The continuously decreasing areas of the dextran tablets should therefore, according to Equation (3), result in slower release rates compared to the tablets in the first category. This is indeed what one can see in Figure 5. By using the suggested model the observed differences between the different types of polymers can be explained.

### 2.5. Relation between release and intrinsic viscosity

**Figure 6:**
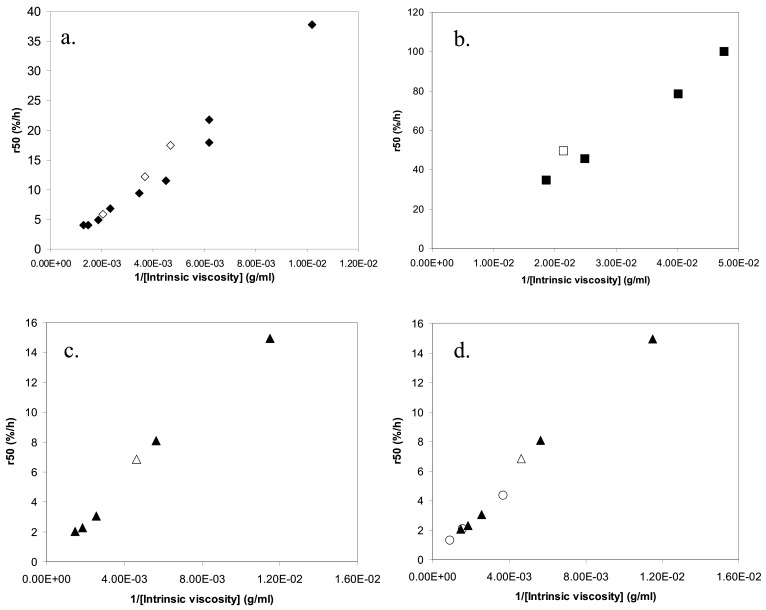
The release rate (r_50_) plotted against the inverse of the intrinsic viscosity of the polymer in the tablet for: (a) PEO, (b) dextran, (c) HPMC 60SH and (d) HPMC 90SH (triangles) together with HPMC 60SH (circles). The filled symbols represent the mixed samples and the unfilled symbols the nonmixed samples.

In our previous studies, we proposed that k_s_, c_s_ and A_s_ in eq 3 should be the same for tablets with same release rate but different polydispersities [7,14]. We also suggested that this would imply that tablets with same release rate would have the same intrinsic viscosity [η], which is a measure of the size of the polymer coil [7,14]. The suggestion was based on the following reasoning. The zero shear viscosity, η_0_, of a polymer solution in a good solvent has repeatedly been found to be a universal function of the reduced concentration c/c*, where c* is the overlap concentration [21] and an estimate of the overlap concentration may be obtained as c* ≈ 1/[η]. From this, it follows that then the viscosity on the surface of the tablet is equal, samples with different degrees of polydispersity should yield the same concentration c_s_ if they have the same value of [η]. If we further assume that k_s_ and A_s_ are insensitive to polydispersity, we obtain from Equation (3) that samples with the same c_s_ should have the same release rate. Indeed, our earlier study showed that the intrinsic viscosity of a PEO sample could, to a good approximation, predict the release rate of the tablet, irrespectively of the polydispersity. In a plot of the release rate, r_50_, versus 1/[η], all data collapsed on a universal relationship. New data obtained in this work, however, giver rise to a relatively large scatter in such plots (Figure 6).

**Figure 7:**
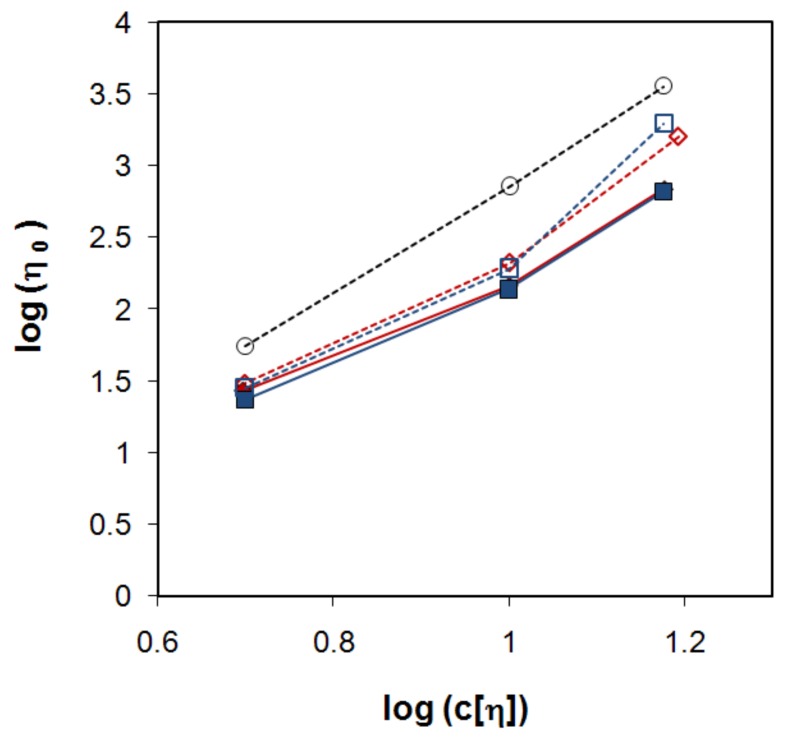
Zero shear viscosity plotted against the reduced concentration on a log-log scale for PEO 0.3 (filled red diamonds), PEO 80:20 (open red diamonds), dextran 500 (filled blue squares) and dextran 50:50 (open blue squares). Since no Newtonian plateau was found for HPMC 60SH 90:10 the viscosity at 1 s^-1^ is included in the plot for comparison. (open grey circles).

**Figure 8:**
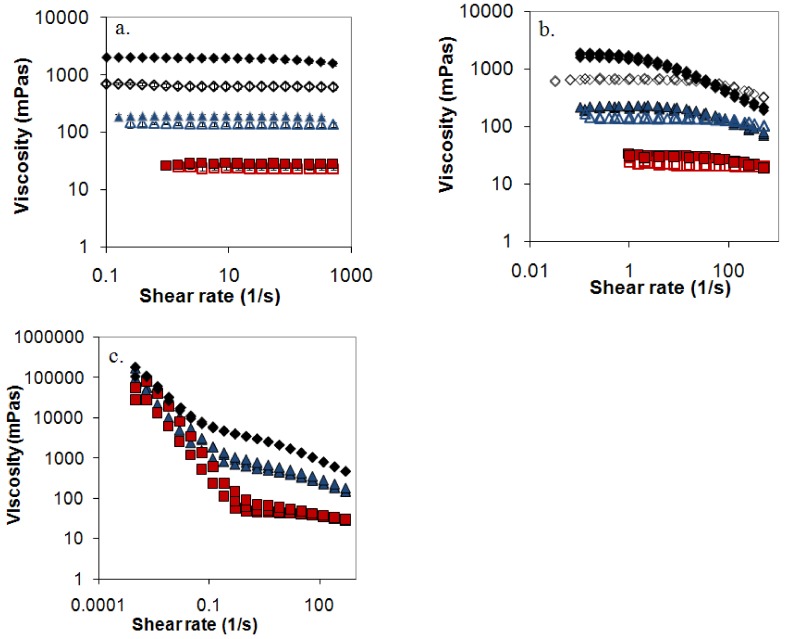
Flow curves (viscosity versus shear rate on a log-log scale) for the measured samples of: (a) dextran 50:50 and dextran 500, (b) PEO 80:20 and PEO 0.3 and (c) HPMC 60SH 90:10 for different reduced concentrations, c [η]=15 (black diamonds), c [η]= 10 (blue triangles) and c [η]=5 (red squares). Filled symbols represent mixed samples and open symbols nonmixed samples.

A central question is if these small deviations in Figure 6 annihilate the proposed model. To test the model the zero shear viscosity was plotted against c[η] (Figure 7), where unfortunately HPMC could not be included in the comparison since its pronounced shear thinning effect made it difficult to estimate the zero shear viscosity (see Figure 8 and Supplementary materials). Figure 7 indeed shows that viscosity curves for mixed and nonmixed samples actually depend differently on c[η]. This indicates that the same viscosity for a nonmixed and a mixed sample would yield differently c[η], and thus equal [η] will give different c_s_ and as a consequence of this different release rates (Equation (3)).

### 2.6. Quantitative aspects

**Figure 9:**
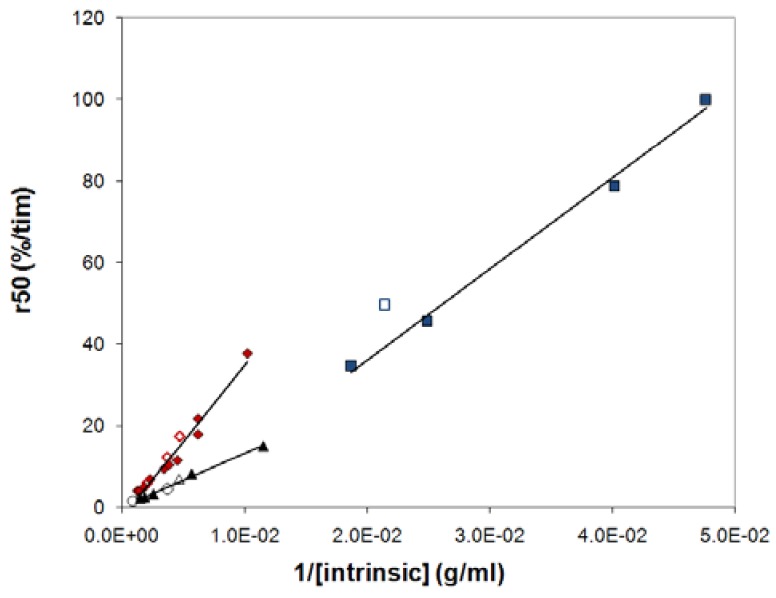
Comparing the relationship between r_50_ and 1/[η] for PEO (red diamonds), dextran (blue squares), HPMC60SH (black triangles) and HPMC90SH (black circles). Filled symbols represent samples in the mixing series, unfilled symbols nonmixed samples. The lines are linear fits to data for the mixed samples, and are included as a guide for the eye.

The many common qualitative features discussed above indicate a common release mechanism for the polymers in the study. Does this also imply quantitative similarities, i.e. do all three polymers obey a single relationship when r_50_ is plotted against 1/[η]? Figure 9 shows that this is not the case. For HPMC there are immediately obvious reasons for a deviation, since HPMC shows significantly shear thinning (see Figure 8) and, presumably for this reason, a significantly different relationship between the low shear viscosity and the overlap parameter than the other mixed polymers. Intramolecular interactions in dilute systems, and intermolecular interactions in semi-dilute systems, of the hydrophobically associating HPMC molecules result in that predictions based on the intrinsic viscosity overestimates the surface concentration (c_s_) of this polymer compared to the other polymers. The slope of the relationship between r_50_ and 1/[η] is therefore expected to be lower for HPMC than for the other polymers. This is indeed the case as can be seen from Figure 9.

**Figure 10:**
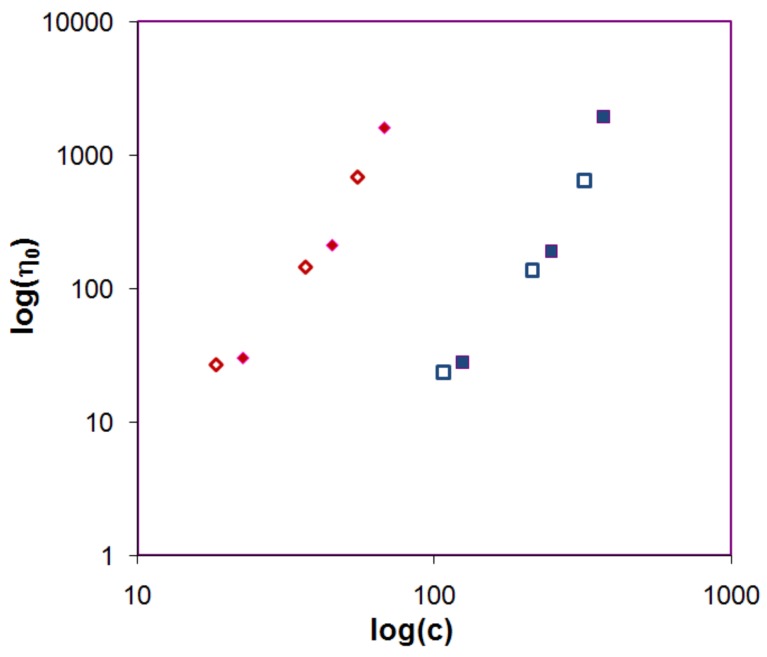
Zero shear viscosity plotted against concentration on a log-log scale for PEO 0.3 (open red diamonds), PEO 80:20 (filled red diamonds), dextran 500 (open blue squares) and dextran 50:50 (filled blue squares).

From Figure 9 it can also be seen that the combined results for mixed PEO and mixed dextran tablets do not give rise to a single master curve, even though they follow the same relationship between the viscosity and the overlap parameter in (see Figure 10 and Supplementary materials). The reason for this deviation should then be attributed to differences in the parameters k_s,50_ and A_s,50_. The rate constant k_s,50_ should depend on the gradient in the chemical potential of water (or, equivalently, the osmotic pressure) in the outermost gel layer, and there is no reason to assume that the osmotic pressure is the same in tablets made of different kinds of polymers. Moreover, if there is a difference in osmotic pressure, the area at t_50_ (A_s,50_) should also vary between the polymer types, even for samples that have the same intrinsic viscosities. Hence, we cannot generally expect all polymers to fall on a single relationship.

## 3. Experimental

### 3.1. Materials

Samples of dextran T70, T500 and T2000 (where the number is indicative of the approximate molecular weight of the dextran grade) were purchased from Amersham Pharmacia, Sweden. HPMC (Metolose) 60SH6, 60SH50, 60SH10000, 90SH100, 90SH4000 and 90SH100000 were kindly supplied by Shin-Etsu, Japan. Here the numbers appearing before and after SH denotate the substitution grade and the viscosity grade, respectively. Thus, 60SH (USP type 2910) and 90SH (USP type 2208) have a methoxy substitution of 28-30 wt% and 19-24 wt%, and a hydroxypropyl substitution of 7-12 wt% and 4-12 wt%, respectively. Polyox WSR N-10 (hereafter referred to as PEO 0.1), Polyox WSR N-750 (hereafter referred to as PEO 0.3), Polyox WSR-1105 (hereafter referred to as PEO 0.9) and Polyox WSR N-60K (hereafter referred to as PEO 2.0) were kindly supplied by Dow, Austria. PEO with a narrow molecular weight distribution (hereafter referred to as PEO 0.3M, where M refers to monodisperse) was purchased from Polymer Laboratories Ltd, (Shropshire, UK). Also used in this work are sodium chloride (MERCK) and sodium azide (BDH Laboratory Supplies).

### 3.2. Tablet preparation

Tablets were made of dextran T70, T500, T2000 and by mixtures of T70 and T2000 at varying ratios. Tablets were also made of HPMC 60SH50, 60SH6, 60SH10000, 90SH100, 90SH4000, 90SH100000 and by mixtures of 60SH6 and 60SH10000 at varying ratios. Mixed tablets are denoted by the mass percentages of the two polymer components, starting with the low molecular weight component. Thus, a sample denoted 90:10 in the dextran series contains 90 wt% T70 and 10 wt% T2000, whereas a sample denoted 90:10 in the 60SH-series contains 90 wt% 60SH6 and 10 wt% 60SH10000. The tablets were made according to a method described earlier [7]. Here follows only a short description of the tablet preparation process. To ensure a homogeneous mixing on the molecular level of the two polymers in mixed samples, 1% w/w aqueous solutions were made of all (mixed or nonmixed) polymer samples. The polymer solutions were freeze-dried using a HETO CD 13-2 freeze-drier. The freeze-dried material was milled before tableting. The tablets were made in a single-punch tableting machine (Kilian SP300, Kilian & Co, GmbH) equipped with 12.0 mm flat-faced punches. The distance between punches was set to 2.0 mm and a compression force of 11.2 ± 0.1 kN. About 315 mg of polymer was introduced into the die and the automatic compression cycle was run. Due to the fluffy nature of the freeze-dried powders, it was difficult to obtain tablets with a uniform tablet weight, especially in the dextran case where the tablet weight could vary by up to 8%.

### 3.3. Moisture content

Since dextran was found to be very hygroscopic, the dextan tablets were dried overnight in a heating cabinet at 60 °C before the dissolution test and the moisture in the HPMC and the dried dextran tablets was measured using a Mettler Toledo HR73 Halogen Moisture Analyzer (Greifensee, Switzerland). One tablet was accurately weighed in the sample pan before slowly heating to 110 °C using the “Gentle Drying” program with a temperature ramp. The weight loss recorded during drying was assumed to be due to loss of moisture. After two hours the value was stable and this value was taken as the moisture content. In the dried dextran tablets the moisture content was between 3.9 and 5.0% and in the HPMC tablets between 4.3 and 5.3% in the 60SH series, and between 6.0 and 8.7% in the 90SH series. The dried tablets in the dextran series used in the dissolution experiments weighed 307 ± 12 mg and the tablets in the HPMC series weighed 315 ± 5 mg.

### 3.4. Release experiment

The release experiments were carried out according to a method described in detail earlier [14]. The release was studied in a USP dissolution apparatus (Dissolutest, Prolabo) equipped with rotating discs. The tablet weight was noted before a tablet was mounted at the centre of a disc using water impermeable glue. The discs were rotated at 100 rpm in the dissolution medium (deionised water, 25 °C). Aliquots (2 mL) were withdrawn from the dissolution medium at different times using a Pharma Test PTFC2 fraction collector. For the rapidly dissolving dextran 100:0 and 90:10 tablets, the initial samples from the dissolution medium were taken manually, using a 2 mL voll pipette, since the fraction collector could not take samples as often as once a minute. At least two tablets of each composition were studied.

### 3.5. Analysis of release samples and determinations of the average molecular weight

The analyses were performed in a similar experimental setup as described before [14], where the polymer content in the release samples was determined using size exclusion chromatography (SEC) combined with refractive index (RI) detection. A multi-angle light scattering (MALS) detector was connected online to the chromatography system providing information of the average molecular weight and molecular weight distribution of the sample. All analyses were performed at room temperature and the flow rate was set to 0.5 ml/min. An aqueous solution of 10 mM NaCl with 0.02% NaN_3_ was used as mobile phase in the HPMC analyses and deionised water with 0.02% NaN_3_ in the dextran analysis. The sample volume injected was 100 μL in both cases. The ASTRA 4.73 software (Wyatt Technology, Santa Barbara, CA) was used for analysis of the obtained RI and MALS chromatograms and refractive index increments, dn/dc, of 0.140 and 0.135 for dextran and HPMC, respectively, was used in the calculations.

The percentage polymer dissolved at each sample time was calculated using the following relation:

(4) $$\%released=\left(\frac{c_{n}\times \left(V_{0}-V_{s}\left(n-1\;\right)\right)+V_{s}\sum_{n=0}^{n-1}c_{n}}{w_{tbl}}\right)\times 100$$

Here c_n_ is the polymer concentration in release sample n, V_0_ is the total volume of the dissolution medium at time t=0, i.e. 500 mL, V_s_ is volume of the release sample, i.e. 2 mL, n is the sample number in the release series and w_tbl_ is the tablet weight. All release profiles were normalized to reach 100% dissolved at the end of the experiment (t=∞).

**Figure 11:**
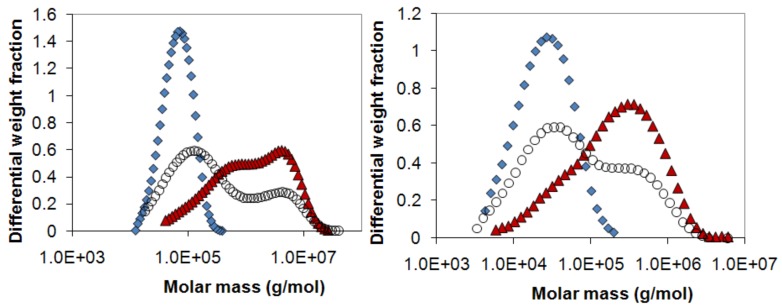
The molecular weight distribution of a mixed 50:50 sample (open circles) together with the pure polymers used in the mixture, (a) dextran T70 (diamonds) and dextran T2000 (triangles) and (b) HPMC 60SH6 (diamonds) and HPMC 60SH10000 (triangles).

The molecular weight distributions for selected dextran and HPMC samples are shown in Figure 11. For both polymers, the high molecular weight polymer sample had a very broad molecular weight distribution, partly containing polymer molecules in the same molecular weight range as the low molecular weight polymer. As a consequence, no baseline separation was obtained between the peaks in a mixed sample. Since no baseline separation was achieved, the retention volume at the minimum between the two polymer peaks for the final release sample in each tablet release series was used as a border line separating two fractions, defined as the high-molecular weight (HMW) and low-molecular weight (LMW) fractions, respectively. The concentration of these fractions could then be determined in each release sample. Note that by this definition, the LMW and HMW fractions are not the same as the fractions of T70 or 60SH6 and T2000 or 60SH10000 originally mixed in the tablet. This is because, for example, both T70 and T2000 contribute with molecules in the LMW range. However, since the molecules from both samples are mixed on a molecular level a polymer molecule of a certain molecular weight behaves in the same way irrespective of which sample it originates from.

### 3.6. Viscometry

The drainage times (t) in an Ubbelohde viscometer at 25.0° C were measured for aqueous solutions of the polymer compositions used in the release experiments as described before [7]. The intrinsic viscosity, [η], was calculated using [22]:

(5) $$[\eta ]\;=\underset{c\to 0}{\lim}\frac{t/t_{0}-1}{c}$$

where t is the drainage time for the PEO solution, t_0_ is the drainage time for the solvent and c is the concentration of the PEO solution.

### 3.7. Rheometry

Flow curves were recorded for selected samples of PEO, dextran and HPMC at various concentrations using an Anton-Paar Physica MCR 500 rheometer. Aqueous solutions of PEO 80:20, PEO 0.3, HPMC 90:10, dextran 50:50 and dextran 500 were prepared at concentrations corresponding to 5/[η], 10/[η] and 15/[η] mg/mL, where [η] was obtained as described above. All measurements were carried out at 25 °C using a cone (diameter 50mm, CP 50-2) and plate geometry. The flow curves were obtained by increasing the shear rate in a logarithmic manner from 10^-3^ s^-1^ to 500 s^-1^. The measuring point duration was decreased logarithmically from 1000 to 10 s and the system was allowed to rest for two minutes before the measurements were started.

## 4. Conclusions

The dissolving polydisperse polymer tablets investigated in this study had several qualitative features in common

- they all developed a gel upon contact with the dissolution medium.
- the shapes of the release curves were similar for mixed and nonmixed samples, with a slower initial rate, an almost linear release rate during the main release period and a slower release rate at the end of the release experiment.
- the polymers showed virtually no fractionation during release.
- the release rate from a tablet of a given polymer could, to a good approximation, be predicted by the average intrinsic viscosity of the polymer sample.

Together this indicates a similar release mechanism for polydisperse PEO, dextran and HPMC tablets dissolving in water. The obtained results support a physical model with the key assumption of a constant viscosity at the tablet surface.

## Figures and Tables

**Table 1:** Characteristics of the tablet compositions used in this study. The average ± one standard deviation (n=3) is given for the number average molecular weight, Mn, the weight average molecular weight Mw, the average intrinsic viscosity [η] and polydispersity index, PI, together with the release rate r_50_ and time when 50% was released t_50_.

Tablet Composition	M_n_/10^5^ (g/mol)	M_w_/10^5^ (g/mol)	PI	[η] (dl/g)	r_50_ (%/h)	t_50_ (h)
**Dextran**						
100:0	0.49±0.01	0.76±0.01	1.56±0.01	0.21	100.0	0.5
90:10	0.38±0.01	3.61±0.26	9.40±0.74	0.25	78.6	0.6
50:50	1.18±0.01	15.2±0.15	13.0±0.72	0.40	45.8	0.9
0:100	4.42±0.15	26.8±0.35	6.06±0.19	0.54	34.7	1.3
T500	2.26±0.04	6.09±0.06	2.64±0.03	0.47	49.6	0.9
**HPMC**						
60SH100:0	0.18±0.01	0.34±0.01	1.87±0.03	0.87	14.9	2.8
60SH90:10	0.17±0.01	0.69±0.01	3.04±1.72	1.77	8.1	6.0
60SH50:50	0.29±0.01	2.07±0.01	7.28±0.13	3.91	3.1	17.4
60SH30:70	0.42±0.01	2.85±0.04	6.75±0.05	5.41	2.3	24.2
60SH0:100	0.80±0.01	3.87±0.12	4.84±0.16	6.31	2.0	29.1
60SH50	0.36±0.01	0.870±0.00	2.38±0.02	2.16	6.8	7.4
90SH100	0.44±0.01	1.19±0.003	2.69±0.02	2.71	4.4	10.5
90SH4000	0.97±0.004	3.01±0.02	3.11±0.02	6.40	2.1	24.3
90SH100000	2.05±0.06	6.14±0.09	3.00±0.13	11.0	1.3	46.8
**PEO**						
PEO 100:0	0.25±0.01^b^	1.22±0.05^a^	4.78^b^	1.0^b^	37.8^a^	1.3^a^
PEO 90:10	0.36±0.01^b^	3.02±0.08^a^	8.39^b^	1.6^b^	21.8^a^	2.3^a^
PEO 88:12	0.40±0.01^c^	3.15±0.03^c^	7.74^c^	1.6	17.9^c^	2.9^c^
PEO 80:20	0.50±0.01^c^	4.35±0.1^c^	8.77^c^	2.2	11.5^c^	4.6^c^
PEO 70:30	0.52±0.01^b^	6.95±0.06^a^	13.4^b^	2.9^b^	9.4^a^	5.8^a^
PEO 50:50	0.64±0.02^b^	12.2±0.33^a^	19.1^b^	4.3^b^	6.8^a^	8.5^a^
PEO 30:70	0.87±0.01^b^	14.5±0.13^a^	16.7^b^	5.4^b^	4.9^a^	11.2^a^
PEO 10:90	1.56±0.06^b^	19.3±0.37^a^	12.4^b^	6.8^b^	4.0^a^	14.1^a^
PEO 0:100	2.34±0.01^b^	21.9±0.07^a^	9.37^b^	7.8^b^	4.0^a^	14.0^a^
PEO 0.3	1.01±0.02^b^	3.9±0.03^b^	3.88^b^	2.7^b^	11.6^b^	4.3^b^
PEO 0.9	1.56±0.09^b^	9.7±0.11^b^	6.28^b^	4.9^b^	5.9^b^	9.4^b^
PEO 0.3M	2.00±0.03^c^	2.34±0.02^c^	1.17^c^	2.1	17.4^c^	2.9^c^

^a^From reference [7]; ^b^From reference [16]; ^c^ From reference [14].

## References

[1] Lapidus H., Lordi N.G. (1966). Some factors affecting the release of a water-soluble drug from a compressed hydrophilic matrix. J. Pharm. Sci..

[2] Lapidus H., Lordi N.G. (1968). Drug release from compressed hydrophilic matrixes. J. Pharm. Sci..

[3] Huber H.E., Dale L.B., Christenson G.L. (1966). Utilization of hydrophilic gums for the control of drug release from tablet formulations I. Disintegration and dissolution behavior. J. Pharm. Sci..

[4] Larsson A., Abrahmsén-Alami S., Juppo A.M., Gad S.C. (2008). Oral Extended Release Formulations. Pharmaceutical Manufacturing Handbook: Production and Processes.

[5] Fukuda M., Peppas N.A., McGinity J. (2006). Properties of sustained release hot-melt extruded tablets containing chitosan and xanthan gum. Int. J. Pharm..

[6] Ju R.T.C., Nixon P.R., Patel M.V. (1995). Drug Release from Hydrophilic Matrixes 1. New Scaling Laws for Predicting Polymer and Drug Release Based on the Polymer Disentanglement Concentration and the Diffusion Layer. J. Pharm. Sci..

[7] Körner A., Larsson A., Piculell L., Wittgren B. (2005). Tuning the polymer release from hydrophilic matrix tablets by mixing short and long matrix polymers. J. Pharm. Sci..

[8] Apicella A., Cappello B., Del Nobile M.A., La Rotonda M.I., Mensitieri G., Nicolais L. (1993). Poly(Ethylene oxide) (PEO) and different molecular weight PEO blends monolithic devices for drug release. Biomaterials.

[9] Gao P., Skoug J.W., Nixon P.R., Ju T.R., Stemm N.L., Sung K.C. (1996). Swelling of hydroxypropyl methylcellulose matrix tablets. 2. Mechanistic study of the influence of formulation variables on matrix performance and drug release. J. Pharm. Sci..

[10] Neau S.H., Chow M.Y., Durrani M.J. (1996). Fabrication and characterization of extruded and spheronized beads containing Carbopol 974P, NF resin. Int. J. Pharm..

[11] Miller-Chou B.A., Koenig J.L. (2003). A review of polymer dissolution. Prog. Polym. Sci..

[12] Narasimhan B., Peppas N.A. (1997). Advances in Polymer Science.

[13] Ueberreiter K., Crank J., Park C.S. (1968). Diffusion in Polymers.

[14] Körner A., Larsson A., Piculell L., Wittgren B. (2005). Molecular information on the dissolution of polydisperse polymers: Mixtures of long and short poly(ethylene oxide). J. Phys. Chem..

[15] Borgquist P., Körner A., Piculell L., Larsson A., Axelsson A. (2006). A model for the drug release from a polymer matrix tablet—effects of swelling and dissolution. J. Control. Release.

[16] Körner A., Andersson Å., Larsson A., Piculell L. (2009). Swelling and polymer erosion for poly(ethylene oxide) tablets of different molecular weights polydispersities. J. Pharm. Sci..

[17] Wittgren B., Wahlund K.-G. (1997). Fast molar mass and size characterization of polysaccharides using asymmetrical flow field flow fractionation – multi angle light scattering. J. Chromatogr. A.

[18] Rajabi-Siahboomi A.R., Bowtell R.W., Mansfield P., Henderson A., Davies M.C., Melia C.D. (1994). Structure and behaviour in hydrophilic matrix sustained release dosage forms: 2. NMR-imaging studies of dimensional changes in the gel layer and core of HPMC tablets undergoing hydration. J. Control. Release.

[19] Clasen C., Kulicke W.-M. (2001). Determination of viscoelastic and rheo-optical material functions of water-soluble cellulose derivatives. Prog. Polym. Sci..

[20] de Gennes P.G. (1979). Scaling Concepts in Polymer Physics.

[21] Morris E.R., Cutler A.N., Ross-Murphy S.B., Rees D.A. (1981). Concentration and shear rate dependence of viscosity in random coil polysaccharide solutions. Carbohydr. Polym..

[22] Atkins P.W. (1998). Physical Chemistry.

